# *catena*-Poly[[dichloridocobalt(II)]-μ-1,2-di-4-pyridylethane-κ^2^ *N*:*N*′]

**DOI:** 10.1107/S1600536808006685

**Published:** 2008-03-14

**Authors:** Zhi-Min Wang

**Affiliations:** a College of Biology and Environmental Engineering, Shuren University, Hangzhou 310015, Zhejiang, People’s Republic of China

## Abstract

In the title compound, [CoCl_2_(C_12_H_12_N_2_)], the Co^II^ atom is coordinated in a tetra­hedral geometry by the N atoms of two different 1,3-di-4-pyridylpropane ligands. The compound adopts a linear chain structure.

## Related literature

For related literature, see: Carlucci *et al.* (2003 ▶); Fujita *et al.* (1998 ▶).
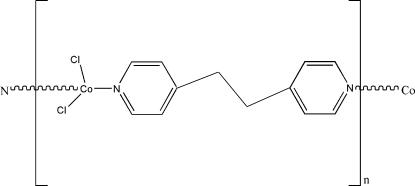


## Experimental

### 

#### Crystal data

[CoCl_2_(C_12_H_12_N_2_)]
*M* *_r_* = 314.07
Triclinic, 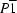

*a* = 5.3979 (17) Å
*b* = 8.806 (3) Å
*c* = 14.018 (4) Å
α = 87.988 (5)°
β = 84.165 (5)°
γ = 84.475 (5)°
*V* = 659.6 (4) Å^3^
*Z* = 2
Mo *K*α radiation
μ = 1.68 mm^−1^
*T* = 298 (2) K
0.27 × 0.21 × 0.18 mm

#### Data collection

Bruker APEXII area-detector diffractometer
Absorption correction: multi-scan (*SADABS*; Sheldrick, 1996 ▶) *T*_min_ = 0.659, *T*_max_ = 0.751
3306 measured reflections
2297 independent reflections
1942 reflections with *I* > 2σ(*I*)
*R*_int_ = 0.022

#### Refinement

*R*[*F* ^2^ > 2σ(*F* ^2^)] = 0.047
*wR*(*F* ^2^) = 0.134
*S* = 1.02
2297 reflections
154 parameters
H-atom parameters constrained
Δρ_max_ = 0.98 e Å^−3^
Δρ_min_ = −1.01 e Å^−3^

### 

Data collection: *APEX2* (Bruker, 2004 ▶); cell refinement: *SAINT* (Bruker, 2004 ▶); data reduction: *SAINT*; program(s) used to solve structure: *SHELXS97* (Sheldrick, 2008 ▶); program(s) used to refine structure: *SHELXL97* (Sheldrick, 2008 ▶); molecular graphics: *SHELXTL* (Sheldrick, 2008 ▶); software used to prepare material for publication: *SHELXTL*.

## Supplementary Material

Crystal structure: contains datablocks I, global. DOI: 10.1107/S1600536808006685/ng2432sup1.cif

Structure factors: contains datablocks I. DOI: 10.1107/S1600536808006685/ng2432Isup2.hkl

Additional supplementary materials:  crystallographic information; 3D view; checkCIF report

## Figures and Tables

**Fig. 1.**
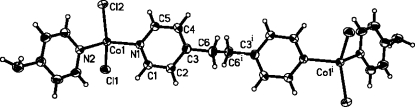
The asymmetric unit of (I), showing 30% probability displacement ellipsoids. (symmetrical code: (i) 1 - x, -y, 1 - z).

## supplementary crystallographic information

### Comment

In recent years, a wide range of 1-D infinite frameworks have been generated by using simple linear bifunctional ligands (Fujita *et al.*, 1998), such as 4,4'-bipyridine (bpy). 1,3-bis(4-pyridyl)propane (bpp) ligand is typical building element for the assembly of infinite architectures. A double-helical chain was synthesized based on transition metal salts and bpp ligand (Carlucci *et al.*, 2003). In this paper, we report the synthesis and crystal structure of the title complex,(I).

As shown in Fig. 1, the complex I is connected to two bpp ligands. The Co^II^ atom in compound I is tetrahedrally coordinatted by two N atoms of two different pyridyl groups and two chloride anions. This coordination mode of cobalt atom is very rare so far. The Co^II^ ions are linked by bpp liagnds and form a zigzag chain. The Co—N bond lengths range from 2.036 (3) to 2.038 (3)Å (Table1). While the Co—Cl bond lengths range from 2.2399 (10) to 2.2484 (11) Å.

### Experimental

CoCl_2_(0.023 g, 0.012 mmol), bpp(0.021 g, 0.013 mmol) were added in a mixed solvent of methanol and benzene, the mixture was heated for ten hours under reflux. During the process stirring and influx were required. The resultant was then filtered to give a pure solution which was infiltrated by diethyl ether freely in a closed vessel, Six weeks later some single crystals was obtained.

### Refinement

The H atoms (pyridine ring) were placed in calculated positions [C*sp*^2^—H = 0.93 Å] and refined using a riding model, with *U*_iso_(H) = 1.2*U*_eq_(C). The maximum peak hole is located on the Co1 with 1.01 Å.

### Crystal data

**Table:**

[CoCl_2_(C_12_H_12_N_2_)]	*Z* = 2
*M**_r_* = 314.07	*F*_000_ = 318
Triclinic, *P*1	*D*_x_ = 1.581 Mg m^−^^3^
Hall symbol: -P 1	Mo *K*α radiation λ = 0.71073 Å
*a* = 5.3979 (17) Å	Cell parameters from 2297 reflections
*b* = 8.806 (3) Å	θ = 1.5–25.1º
*c* = 14.018 (4) Å	µ = 1.68 mm^−^^1^
α = 87.988 (5)º	*T* = 298 (2) K
β = 84.165 (5)º	Block, pink
γ = 84.475 (5)º	0.27 × 0.21 × 0.18 mm
*V* = 659.6 (4) Å^3^	

### Data collection

**Table:**

Bruker APEXII area-detector diffractometer	2297 independent reflections
Radiation source: fine-focus sealed tube	1942 reflections with *I* > 2σ(*I*)
Monochromator: graphite	*R*_int_ = 0.022
*T* = 298(2) K	θ_max_ = 25.1º
φ and ω scans	θ_min_ = 1.5º
Absorption correction: multi-scan(SADABS; Sheldrick, 1996)	*h* = −4→6
*T*_min_ = 0.659, *T*_max_ = 0.752	*k* = −9→10
3306 measured reflections	*l* = −16→16

### Refinement

**Table:**

Refinement on *F*^2^	Secondary atom site location: difference Fourier map
Least-squares matrix: full	Hydrogen site location: inferred from neighbouring sites
*R*[*F*^2^ > 2σ(*F*^2^)] = 0.047	H-atom parameters constrained
*wR*(*F*^2^) = 0.134	*w* = 1/[σ^2^(*F*_o_^2^) + (0.1008*P*)^2^] where *P* = (*F*_o_^2^ + 2*F*_c_^2^)/3
*S* = 1.03	(Δ/σ)_max_ = 0.001
2297 reflections	Δρ_max_ = 0.98 e Å^−^^3^
154 parameters	Δρ_min_ = −1.01 e Å^−^^3^
Primary atom site location: structure-invariant direct methods	Extinction correction: none

### Special details

**Table:**

Geometry. All e.s.d.'s (except the e.s.d. in the dihedral angle between two l.s. planes) are estimated using the full covariance matrix. The cell e.s.d.'s are taken into account individually in the estimation of e.s.d.'s in distances, angles and torsion angles; correlations between e.s.d.'s in cell parameters are only used when they are defined by crystal symmetry. An approximate (isotropic) treatment of cell e.s.d.'s is used for estimating e.s.d.'s involving l.s. planes.
Refinement. Refinement of *F*^2^ against ALL reflections. The weighted *R*-factor *wR* and goodness of fit *S* are based on *F*^2^, conventional *R*-factors *R* are based on *F*, with *F* set to zero for negative *F*^2^. The threshold expression of *F*^2^ > σ(*F*^2^) is used only for calculating *R*-factors(gt) *etc*. and is not relevant to the choice of reflections for refinement. *R*-factors based on *F*^2^ are statistically about twice as large as those based on *F*, and *R*- factors based on ALL data will be even larger.

### Fractional atomic coordinates and isotropic or equivalent isotropic displacement parameters (Å^2^)

**Table:**

	*x*	*y*	*z*	*U*_iso_*/*U*_eq_	
Co1	0.88487 (8)	0.54658 (4)	0.74750 (3)	0.0386 (2)	
Cl1	1.00798 (18)	0.70640 (10)	0.62711 (6)	0.0535 (3)	
Cl2	1.14897 (18)	0.42183 (11)	0.84306 (6)	0.0533 (3)	
N1	0.7275 (5)	0.3806 (3)	0.68363 (19)	0.0403 (6)	
N2	0.6308 (5)	0.6763 (3)	0.8353 (2)	0.0421 (7)	
C1	0.5435 (7)	0.4131 (4)	0.6281 (3)	0.0456 (8)	
H1	0.4851	0.5151	0.6200	0.055*	
C2	0.4336 (7)	0.3066 (4)	0.5819 (3)	0.0489 (9)	
H2	0.3053	0.3362	0.5438	0.059*	
C3	0.5184 (7)	0.1528 (4)	0.5933 (2)	0.0466 (8)	
C4	0.7079 (8)	0.1187 (4)	0.6500 (3)	0.0564 (10)	
H4	0.7711	0.0176	0.6585	0.068*	
C5	0.8076 (7)	0.2329 (4)	0.6950 (3)	0.0518 (9)	
H5	0.9343	0.2062	0.7344	0.062*	
C6	0.4098 (7)	0.0307 (4)	0.5416 (3)	0.0536 (9)	
H6A	0.2547	0.0730	0.5176	0.064*	
H6B	0.3720	−0.0523	0.5864	0.064*	
C7	0.4596 (8)	0.7756 (4)	0.7979 (3)	0.0561 (10)	
H7	0.4611	0.7858	0.7316	0.067*	
C8	0.2815 (8)	0.8629 (5)	0.8552 (3)	0.0628 (11)	
H8	0.1652	0.9304	0.8270	0.075*	
C9	0.2745 (7)	0.8507 (4)	0.9545 (3)	0.0508 (9)	
C10	0.4501 (8)	0.7480 (5)	0.9904 (3)	0.0582 (10)	
H10	0.4527	0.7354	1.0565	0.070*	
C11	0.6216 (7)	0.6636 (4)	0.9308 (3)	0.0532 (9)	
H11	0.7372	0.5942	0.9578	0.064*	
C12	0.0852 (8)	0.9406 (4)	1.0232 (3)	0.0610 (11)	
H12A	−0.0164	0.8697	1.0598	0.073*	
H12B	0.1746	0.9903	1.0680	0.073*	

### Atomic displacement parameters (Å^2^)

**Table:**

	*U*^11^	*U*^22^	*U*^33^	*U*^12^	*U*^13^	*U*^23^
Co1	0.0492 (3)	0.0344 (3)	0.0329 (3)	−0.0032 (2)	−0.0083 (2)	−0.00010 (19)
Cl1	0.0644 (6)	0.0466 (5)	0.0484 (5)	−0.0056 (4)	−0.0054 (4)	0.0137 (4)
Cl2	0.0599 (6)	0.0565 (6)	0.0444 (5)	−0.0006 (4)	−0.0178 (4)	0.0065 (4)
N1	0.0491 (16)	0.0361 (15)	0.0366 (15)	−0.0040 (12)	−0.0079 (12)	−0.0013 (11)
N2	0.0495 (16)	0.0389 (15)	0.0389 (15)	−0.0014 (12)	−0.0116 (12)	−0.0018 (12)
C1	0.055 (2)	0.0361 (18)	0.046 (2)	−0.0001 (15)	−0.0103 (16)	−0.0003 (15)
C2	0.055 (2)	0.049 (2)	0.045 (2)	−0.0050 (16)	−0.0140 (16)	−0.0012 (16)
C3	0.057 (2)	0.044 (2)	0.0397 (19)	−0.0111 (16)	−0.0043 (16)	−0.0026 (15)
C4	0.076 (3)	0.0332 (19)	0.063 (3)	−0.0038 (17)	−0.020 (2)	−0.0002 (17)
C5	0.064 (2)	0.039 (2)	0.055 (2)	−0.0021 (16)	−0.0222 (18)	−0.0003 (16)
C6	0.066 (2)	0.049 (2)	0.049 (2)	−0.0170 (18)	−0.0087 (18)	−0.0057 (17)
C7	0.070 (2)	0.060 (2)	0.0354 (19)	0.0119 (19)	−0.0096 (17)	0.0024 (17)
C8	0.068 (3)	0.065 (3)	0.050 (2)	0.023 (2)	−0.0088 (19)	0.0051 (19)
C9	0.060 (2)	0.047 (2)	0.043 (2)	0.0028 (17)	−0.0025 (16)	−0.0014 (16)
C10	0.070 (3)	0.065 (3)	0.0367 (19)	0.012 (2)	−0.0080 (17)	−0.0022 (17)
C11	0.061 (2)	0.053 (2)	0.045 (2)	0.0080 (17)	−0.0085 (17)	−0.0017 (17)
C12	0.070 (3)	0.061 (3)	0.047 (2)	0.012 (2)	−0.0005 (19)	0.0018 (18)

### Geometric parameters (Å, °)

**Table:**

Co1—N1	2.036 (3)	C5—H5	0.9300
Co1—N2	2.038 (3)	C6—C6^i^	1.521 (7)
Co1—Cl2	2.2399 (10)	C6—H6A	0.9700
Co1—Cl1	2.2484 (11)	C6—H6B	0.9700
N1—C1	1.327 (4)	C7—C8	1.379 (5)
N1—C5	1.340 (4)	C7—H7	0.9300
N2—C11	1.335 (5)	C8—C9	1.390 (5)
N2—C7	1.343 (5)	C8—H8	0.9300
C1—C2	1.370 (5)	C9—C10	1.368 (5)
C1—H1	0.9300	C9—C12	1.514 (5)
C2—C3	1.397 (5)	C10—C11	1.364 (5)
C2—H2	0.9300	C10—H10	0.9300
C3—C4	1.362 (5)	C11—H11	0.9300
C3—C6	1.512 (5)	C12—C12^ii^	1.500 (8)
C4—C5	1.385 (5)	C12—H12A	0.9700
C4—H4	0.9300	C12—H12B	0.9700
			
N1—Co1—N2	112.50 (11)	C3—C6—C6^i^	111.5 (4)
N1—Co1—Cl2	105.20 (8)	C3—C6—H6A	109.3
N2—Co1—Cl2	106.04 (8)	C6^i^—C6—H6A	109.3
N1—Co1—Cl1	105.09 (9)	C3—C6—H6B	109.3
N2—Co1—Cl1	104.94 (9)	C6^i^—C6—H6B	109.3
Cl2—Co1—Cl1	123.22 (4)	H6A—C6—H6B	108.0
C1—N1—C5	116.8 (3)	N2—C7—C8	121.8 (3)
C1—N1—Co1	121.9 (2)	N2—C7—H7	119.1
C5—N1—Co1	121.3 (2)	C8—C7—H7	119.1
C11—N2—C7	117.5 (3)	C7—C8—C9	120.5 (4)
C11—N2—Co1	122.3 (2)	C7—C8—H8	119.7
C7—N2—Co1	120.2 (2)	C9—C8—H8	119.7
N1—C1—C2	124.5 (3)	C10—C9—C8	116.2 (3)
N1—C1—H1	117.8	C10—C9—C12	119.4 (3)
C2—C1—H1	117.8	C8—C9—C12	124.4 (4)
C1—C2—C3	118.7 (3)	C11—C10—C9	121.0 (4)
C1—C2—H2	120.7	C11—C10—H10	119.5
C3—C2—H2	120.7	C9—C10—H10	119.5
C4—C3—C2	117.2 (3)	N2—C11—C10	122.9 (3)
C4—C3—C6	121.9 (3)	N2—C11—H11	118.5
C2—C3—C6	120.9 (3)	C10—C11—H11	118.5
C3—C4—C5	120.7 (3)	C12^ii^—C12—C9	115.1 (4)
C3—C4—H4	119.6	C12^ii^—C12—H12A	108.5
C5—C4—H4	119.6	C9—C12—H12A	108.5
N1—C5—C4	122.1 (3)	C12^ii^—C12—H12B	108.5
N1—C5—H5	118.9	C9—C12—H12B	108.5
C4—C5—H5	118.9	H12A—C12—H12B	107.5
			
N2—Co1—N1—C1	58.9 (3)	C6—C3—C4—C5	178.7 (4)
Cl2—Co1—N1—C1	173.9 (3)	C1—N1—C5—C4	1.1 (6)
Cl1—Co1—N1—C1	−54.7 (3)	Co1—N1—C5—C4	−178.1 (3)
N2—Co1—N1—C5	−121.9 (3)	C3—C4—C5—N1	−1.4 (7)
Cl2—Co1—N1—C5	−6.9 (3)	C4—C3—C6—C6^i^	−72.0 (6)
Cl1—Co1—N1—C5	124.5 (3)	C2—C3—C6—C6^i^	105.6 (5)
N1—Co1—N2—C11	106.3 (3)	C11—N2—C7—C8	0.6 (6)
Cl2—Co1—N2—C11	−8.2 (3)	Co1—N2—C7—C8	178.6 (3)
Cl1—Co1—N2—C11	−140.0 (3)	N2—C7—C8—C9	0.2 (7)
N1—Co1—N2—C7	−71.5 (3)	C7—C8—C9—C10	−0.6 (7)
Cl2—Co1—N2—C7	174.0 (3)	C7—C8—C9—C12	−179.5 (4)
Cl1—Co1—N2—C7	42.2 (3)	C8—C9—C10—C11	0.3 (6)
C5—N1—C1—C2	−0.5 (6)	C12—C9—C10—C11	179.2 (4)
Co1—N1—C1—C2	178.8 (3)	C7—N2—C11—C10	−1.0 (6)
N1—C1—C2—C3	0.1 (6)	Co1—N2—C11—C10	−178.9 (3)
C1—C2—C3—C4	−0.3 (6)	C9—C10—C11—N2	0.6 (7)
C1—C2—C3—C6	−178.0 (3)	C10—C9—C12—C12^ii^	175.1 (5)
C2—C3—C4—C5	1.0 (6)	C8—C9—C12—C12^ii^	−6.1 (8)

Symmetry codes: (i) −*x*+1, −*y*, −*z*+1; (ii) −*x*, −*y*+2, −*z*+2.
